# Transfer learning of clinical outcomes from preclinical molecular data, principles and perspectives

**DOI:** 10.1093/bib/bbac133

**Published:** 2022-04-23

**Authors:** Axel Kowald, Israel Barrantes, Steffen Möller, Daniel Palmer, Hugo Murua Escobar, Anne Schwerk, Georg Fuellen

**Affiliations:** 1 Institute for Biostatistics and Informatics in Medicine and Ageing Research, Rostock University Medical Center, Rostock, Germany; 2 Department of Medicine, Clinic III, Hematology, Oncology, Palliative Medicine, Rostock University Medical Center, Rostock, Germany; 3 CENTOGENE GmbH, Am Strande 7, Rostock, Germany; 4 Centre for Transdisciplinary Neurosciences Rostock, Research Focus Oncology and Ageing of Individuals and Society, Interdisciplinary Faculty, Rostock, Germany

**Keywords:** transfer learning, shared denominators, biomarkers, transductive transfer learning, inductive transfer learning, unsupervised transfer learning

## Abstract

Accurate transfer learning of clinical outcomes from one cellular context to another, between cell types, developmental stages, omics modalities or species, is considered tremendously useful. When transferring a prediction task from a source domain to a target domain, what counts is the high quality of the predictions in the target domain, requiring states or processes common to both the source and the target that can be learned by the predictor reflected by shared denominators. These may form a compendium of knowledge that is learned in the source to enable predictions in the target, usually with few, if any, labeled target training samples to learn from. Transductive transfer learning refers to the learning of the predictor in the source domain, transferring its outcome label calculations to the target domain, considering the same task. Inductive transfer learning considers cases where the target predictor is performing a different yet related task as compared with the source predictor. Often, there is also a need to first map the variables in the input/feature spaces and/or the variables in the output/outcome spaces. We here discuss and juxtapose various recently published transfer learning approaches, specifically designed (or at least adaptable) to *predict clinical (human in vivo) outcomes based on preclinical (mostly animal-based) molecular data*, towards finding the right tool for a given task, and paving the way for a comprehensive and systematic comparison of the suitability and accuracy of transfer learning of clinical outcomes.

## Introduction

‘Translation’ in biomedicine can often be fostered by the transfer of knowledge from a source domain to a target domain. In biomedicine, more and more molecular data have become available, with the potential to help with diagnosis, prognosis as well as treatment development, selection and monitoring. Such data may be available as a source of knowledge from, e.g. human blood, cell cultures or animals, but they are often not available for the human target tissue, e.g. an inoperable tumor or brain tissue. More generally, translating (i.e. transferring) insights from models, across tissues or species, or to the human *in vivo* situation has been a long-term challenge [1–3]. Given cancer cell line or advanced tumor model data, can we predict human treatment success of cancer drugs? Given toxicology data from rabbits, rodents or dogs, can we predict drug-mediated tolerance or toxicity in humans? For this narrative review, we collected a diverse set of recently published transfer learning tools, all with application examples from biomedicine. We enable their comparison in terms of their application domain(s), input, output and methodology, and we give some guidance regarding the choice of these tools for a specific learning task.

Easily accessible molecular data for the model (or proximate) situations of blood, cell cultures or animal experiments are becoming more abundant in biomedicine often based on high-throughput technologies (omics) [4, 5]. Organoids and multi-organ-chip models are also sources of additional model data with increasing relevance and usage [6]. As a first transfer learning step, we may wish to work on the successful transfer of conserved *molecular processes and associated biomarkers across data sets*. On that basis, we may be able to process and organize the model data to enable a successful transfer of insights about *outcomes*, referring to the human *in vivo* situation. Alternatively, outcome predictors may be transferred directly from the model to humans. For the example of predicting intervention outcomes in humans, knowledge of processes and biomarkers then enables clinical trials ‘enriched’ with likely responders, or with co-treatment of non-responders, to counter insufficient effect (or overly-strong side-effects). Even better, we would like to estimate the chances of success of a clinical trial a priori. In turn, clinical insights can inform preclinical work (``reverse translation'') [1] and transfer learning can thus be of high value also in the direction from the clinical to the preclinical world. 

Accurate transfer is so valuable because insights for human *in vivo* data are so hard to obtain; clinical outcomes are usually expensive to establish, limited in numbers and cannot easily be shared with other researchers due to data protection regulations. The constrained accessibility of most human tissues implies that detailed clinical data, mechanistic understanding and useful biomarkers cannot easily be obtained. Moreover, biomedicine is a ‘rich’ and highly heterogeneous discipline, where most molecular processes, including their causal influence on phenotypes, are rarely conserved: they differ by cellular context (*in vitro* versus *ex vivo* versus *in vivo*, or across tissues), cell type, developmental stage, molecular entity (omics modality) and species, and even by the genetics of the individual cells, animals or humans from which the data are gathered. Case in point are the on-going discussions about the similarity or dissimilarity of immune responses in mouse versus human (e.g. [7] versus [8] and the discussion in [9]). Thus, the context-dependency of cellular responses and of their high-level phenotypic implications is significant. It is one of several causes of what is called the ‘reproducibility crisis’ and it can cause translational failures. Also, correlation and causality are not easy to discern. While correlative relationships are sometimes sufficient (e.g. for a biomarker to be predictive), causal relationships are telling us much more about the information flow that starts molecularly and ends up in generating the high-level outcome phenotypes that are of ultimate clinical interest [10]. Transfer learning, if based on causal relationships, can thus be expected to be more successful in general. In any case, we must aim for accurate transfer learning to the best of our abilities.

With the introduction of high-throughput technologies such as genotype–phenotype association mapping in clinical cohorts (GWAS and polygenic risk scores) and gene expression measurements by microarray or RNAseq (transcriptomics), we can investigate molecular mechanisms, including intervention effects, side effects and other changes in time. These investigations are becoming more and more thorough, *in vitro* (for human and animal models) as well as *in vivo* (mostly for animal). However, the molecules that we can measure as potential markers are just a glimpse of the intricate *in vivo* situation, whereby the measurements for one molecular modality (such as mRNA) are in a complex relationship to the measurements of another (such as proteins) [11]. Furthermore, the available datasets often differ significantly in quality and granularity, show large batch effects from one measurement to the other and lack comprehensive sample descriptions including detailed source specifications as well as adequate sample (pre)processing details, impeding straightforward comparability. As described, clinical molecular data (from human *in vivo* studies) are scarce mainly due to limited tissue availability and data protection issues, even though blood may be more readily available and genetic information is readily obtainable even though it may not be easy to share. One particular consideration in transfer learning is the context-dependency and consistency of data in the source and target domains, as the authors of AITL [12] pointed out. Domain discrepancies can include differences in extracted features, due to divergent biology, e.g. cell types versus tissues, and they can arise from differences in effect measurements, e.g. continuous versus binary outcomes. Therefore, training a computational model on cell lines and testing it on patients violates the i.i.d. assumption that train and test data are from the same distribution [12]. Other frequently encountered source versus target domain differences hindering transfer learning are the species gap and the complex relationships between the molecular entities, e.g. mRNA versus proteins, that may constitute the source versus target domain.

### Transfer learning, terminology and examples

By default, we follow [12] in adopting the terminology of Pan and Yang [13], which is a widely cited review and reference in the field of transfer learning. In their paper, as in the more recent review of Zhuang *et al.* [14], transfer learning is defined in terms of source and target domains (of features with probability distributions associated with these), as well as source and target tasks (mapping features to labels using predictors) so that the predictor in the target domain is based on training examples from the source domain, which depends on the extraction of shared denominators to enable the transfer into the target domain. In a simple case, that means to learn a predictor for the source task in the source domain, and then to just use it, after matching the input/output variables, as the predictor for the target task in the target domain. For any real transfer to take place, the source and target domain, or the source and target tasks, must of course be distinct but contain shared denominators to be transferred. According to Pan and Yang, such shared denominators can be at the level of (i) weighted instances, (ii) feature representations, (iii) model parameters or (iv) logical relationships—this review will not discuss these in detail for the transfer learning approaches that are presented, but it will simply refer to these as ‘shared denominators’. Frequently in the literature, shared feature representations are known as ‘latent variables’ (LVs), and we will specifically mention these when describing some of the tools.

Considering the transfer gaps just described, a sufficient degree of similarity, or conservation, of the source and the target is the key to accurate transfer learning from a well-sampled source domain to an under-sampled or unknown target domain. Considering the predictors, which map the input features to output labels, shared denominators are calculated by these predictors in the source domain, and their similar role in the target is a necessity for transfer learning to succeed. After learning in the source domain, the predictor contains knowledge about this domain. If the prediction is based on a neural net, this knowledge is represented by the weights and the biases of the neural net based on the input features provided and learned in the source domain. As an example, gene expression to phenotype relationships may be learned by a neural net predictor in one species and then transferred to another species. Then, the weights and biases are supposed to reflect how gene expression maps to phenotypes. If the mapping is sufficiently similar in the two domains, then the predictor can be applied successfully to the target domain. If the shared denominators refer to features, they can be thought of as low-dimensional representations of the input data that reflect the major features for a given prediction task. As an analogy, principal component analysis (PCA) derives these as eigenvectors of a matrix of an all-against-all covariance analysis. Often, a compendium of shared denominators is learned from the source data to enable transfer learning, and the example of ‘MultiPlier’ [15] as a compendium of LVs based on analyzing gene expression data using matrix factorization will be presented below.

Generally, transfer learning is defined as belonging to transductive, inductive or unsupervised approaches. The *transductive* flavor of transfer learning (sometimes also called heterogeneous transfer learning) entails different but related source and target domains, but the tasks are the same and the target domain does not need to have any labeled samples [13], so that the predictor that uses the shared denominators from the source domain can be successfully applied to the target domain. The prediction/classification task must be the same in the source and target, as it was for the simple neural net example discussed above, which learned to map gene expression to phenotype data. The *inductive* flavor of transfer learning considers tasks that are different yet ‘related’, and the target domain must include labeled samples [13]. While the tasks are different, the source and target domains (and the marginal distributions of the data) are supposed to be the same. There is usually no formal definition of the relatedness of the tasks; the ‘proof is in the pudding’, that is, accurate transfer in terms of correct predictions/classifications is the indicator of sufficient relatedness of the task in the source and the task in the target domain. At least, a few labeled samples are needed in the target domain, so that the knowledge about the shared denominators can be grounded to some true relations in the target domain. Modifying our simple example from above, about mapping gene expression to phenotype, if the phenotype is morbidity in one species and mortality in the other species, the task is different yet related and inductive transfer learning may be applied. We also consider the entirely *unsupervised* flavor of transfer learning, where the source and target domains are different but related and the tasks are also different but related, and none of the samples contain any labels, neither in the source nor in the target domain. In this case, the underlying shared denominators are employed to transfer, from the source to the target, information useful for clustering, for better feature representation or for dimensionality reduction [13]; see the examples given below.

In this review, we specifically consider preclinical source and clinical target domains, towards clinical outcome prediction, as well as molecular omics data as the dominant sample features that are input for the predictors. As an aside, the notion of pre-training is sometimes used to refer to the *first step* of transfer learning, as described by [14] for the example of using neural nets to learn images. Pre-training with a large compendium of images then avoids initializing the neural net with random weights, and it prepares for learning a more specific set of images; it can improve accuracy (by avoiding overfitting) as well as execution time of the final classification [16, 17].

### Application of transfer learning

**Figure 1:**
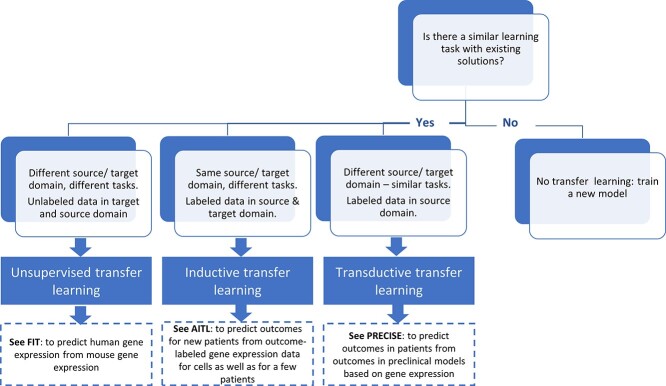
How to select a transfer learning methodology for a given task. This given task is the ‘learning task with existing solutions’ and it refers to the source domain, see also Table 1 and [13]. For the examples (FIT, AITL and PRECISE), please see Table 1.

In Figure 1, we provide a simple flowchart regarding the selection process for the different transfer learning approaches, as detailed in the previous section. Application areas of transfer learning feature on one hand a target domain where few or no solutions are given, and on the other hand, a source domain where many more solutions are available. Thus, for a user faced with a task that they think could be solved by transfer learning, the first and foremost goal is to answer the question: ‘Is there a problem domain, and a learning task in that domain, different from what we are looking at, where there are already (many) solutions that may be of relevance to the task we have?’. If yes, the second question then is: ‘Is our task a case for inductive, transductive or unsupervised transfer?’. For this second question, Figure 1 summarizes how to select among the approaches for transfer learning. In principle, any need to learn labels (such as diagnoses, or outcomes, be they a disease prognosis or the prediction of the success or failure of an intervention) requires a supervised approach, which may be inductive or transductive, as both use labeled source domain data. Furthermore, an inductive method will be needed if the learning tasks are different, comparing the source to the target; in this case, some labeled samples are required for the target domain. For example, in the case of AITL [12], based on gene expression data as input, the source task is to predict the IC50 (a quantity), and the target task is to predict patient response (yes or no). If the tasks are the same, a transductive method would be sufficient, and ‘domain adaptation’, e.g. by relabeling, may be the way to go; furthermore, no labeled samples are required for the target domain. For example, in case of the semi-supervised transfer learning of [9], the task is phenotype/outcome prediction for human (the target domain), transferred from the same task for mouse (the source domain), and domain adaptation entails the mapping of homologous genes. Finally, if no labels need to be learned, our user may explore whether unsupervised transfer learning, e.g. of association or enrichment data, is possible and useful.

### Recent examples of transfer learning

In the biomedical application areas we consider here, the distinct domains or tasks to be transferred reflect different cellular contexts (*in vitro* versus *ex vivo* versus *in vivo*), tissues or cell types, omics modalities or species. Due to the great need for improved clinical translation, many studies investigated ‘preclinical’ source data and ‘clinical’ target data.

In Table 1, we collected some recently published representative examples without claim to completeness. We aimed for high-level descriptions while keeping formulas at a minimum. We describe the source domain, frequently also known as the ‘background model’ or ‘compendium’, and the target domain. Further, we describe the input and output of the predictor that is learned, the kind of transfer learning methodology employed, labeling it as ‘transductive’, ‘inductive’ or ‘unsupervised’, following [13], and we describe whether the method was compared with others (and if so, how it performed). This table lays the foundation for finding the right tool for a user’s task by conceptual similarity matching of the user’s task to the entries in the table. Optimally, this matching follows a principled approach, considering the kind of transfer learning (Figure 1). It can also be seen as the *starting point* for a comprehensive and systematic comparison of state-of-the-art transfer learning methods, considering a variety of application scenarios. In the following, we give a textual description of the examples in Table 1, providing details not fitting into the table.

**Table 1:** Transfer learning, tools and techniques

Name/acronym, reference	Source domain	Target domain	Input of the predictors	Output of the predictors	Transfer method; regression or classification task?	Availability, advantages and disadvantages (results/accomplishments)
Semisupervised transfer learning [9]	Application-area-specific mouse phenotype-outcome-labeled gene expression data	Human gene expression data	Human gene expression data	Human phenotype data (and subsequently DEGs and enriched pathways inferred from these)	*Transductive*: supervised modeling (mouse) amended iteratively by semi-supervised retraining (adding unlabeled human data); classification task	Matlab code available from www.mathworks.com/matlabcentral/fileexchange/69718-semisupervised-learning-functions. Compared favorably in various metrics to different machine learning methods like kNN, SVM and RF
XGSEA [18]	GO (or similar) gene sets and enrichment scores, e.g. from mouse or zebrafish	GO (or similar) gene sets and enrichment scores, e.g. from human	Gene expression data from source species used to calculate enrichment scores	Gene sets significantly associated in target species	*Transductive*: domain adaptation followed by prediction of significantly associated gene sets; regression task: logistic on *P*-values, linear on enrichment scores or linear on positive and negative enrichment scores separately	Code available at https://github.com/LiminLi-xjtu/XGSEA Compared favorably in various metrics to three naïve methods also proposed in the paper. XGSEA produced a smaller but more focused list of significant GO terms in the reported case study than the best performing naïve method. Depending on the needs of a study this could be an advantage or disadvantage to further interpretation
FIT [19]	Precompiled datasets of mouse gene expression	Precompiled datasets of human gene expression	Mouse gene expression	Human gene expression for matching condition, genes with high effect size	Unsupervised (dimensionality reduction): gene-level lasso regression; follow-up classification task to identify high-effect genes	Available at http://www.mouse2man.org; including pre-test for transferability; compared favorably to predictions based only on mouse data
Translatable components regression (TransComp-R) [20]	Human gene expression data (pretreatment), human drug response data	Mouse proteomics data	Human gene expression (pretreatment) and drug response data (the latter are given, not to be predicted)	Mouse proteins (and corresponding pathway enrichments) with association to human drug response	*Unsupervised (feature representation):* PCA-based regression	Matlab code available from https://de.mathworks.com/matlabcentral/fileexchange/77987-transcompr. Experimental verification of a gene predicted to be involved in resistance to treatment; apparently no other benchmarking
Pathway RespOnsive GENes (PROGENy) [21] and Discriminant Regulon Expression Analysis (DoRothEA) [22]	Two curated resources of footprint pathway perturbations (PROGENy), and another of footprint regulons (transcription factor—target interactions in DoRothEA) from human data, and human–mouse orthologs	The mouse equivalent of the source	Mouse gene expression data	Mouse pathway activity (PROGENy) or transcription factor activity and enrichment (DoRothEA)	*Transductive*: supervised prediction of mouse pathways (PROGENy) and regulons (DoRothEA); regression task	Both tools are available as R (Bioconductor) and python packages; for usage examples see https://github.com/saezlab/transcriptutorial; no benchmarking is described by the authors
Adversarial Inductive Transfer Learning (AITL) [12]	*In vitro* (cell line) gene expression and quantitative outcome (IC50) data	*In vivo* (patient) gene expression and qualitative outcome (yes/no) data	*In vitro* gene expression data (GDSC)	*In vivo* outcomes (TCGA)	*Inductive*: adversarial domain adaptation and multi-task learning (predicting outcomes for both source and target) using deep neural nets; classification task in the target domain	Code available at https://github.com/hosseinshn/AITL; performance benchmarked against six other methods (see main text) and found to perform best
Patient Response Estimation Corrected by Interpolation of Subspace Embeddings (PRECISE) [24]	Gene expression data from preclinical models (cell lines, patient-derived xenografts) and drug response	Human gene expression data	Human gene expression data	Human drug response	*Transductive*: similarity-based identification of shared mechanisms between large datasets from preclinical models and a small number of human samples, focused on cancer; regression task	Available as python package,; example protocols provided as Jupyter notebooks; see https://github.com/NKI-CCB/PRECISE; outperforming two state-of-the-art approaches (ridge regression on either the raw or ComBat corrected gene expression data) on retrieving associations between known biomarkers and drug responses
Transfer variational autoencoder, trVAE [30]	Gene expression data (cell line) or image data (or similar) under a specific (first) condition	Gene expression data or image data (or similar) under a different (second) condition	Data under the first condition and a label specifying the second condition	Data transformed to the second condition	*Transductive:* based on an autoencoder neural net; regression-like task when applied to expression data	Available from https://github.com/theislab/trvae_reproducibility; benchmarked against six other tools (see main text) and found to perform best
MultiPlier [15]	Preprocessed disease-related datasets of human gene expression, highlighting LVs (characteristic patterns of correlated genes)	Human (rare disease) gene expression data	Human (rare disease) gene expression data	Characteristic expression patterns of correlated genes	*Unsupervised (feature representation):* constrained matrix factorization highlighting LVs, then projection of input into latent space; neither regression nor classification	PLIER is available at https://github.com/wgmao/PLIER; MultiPlier is available from https://github.com/greenelab/multi-plier with a summary of additional dependencies also described in the accompanying paper. A docker image is provided to reproduce the analyses; no benchmarking is described by the authors

*Semisupervised Transfer Learning*, as described by [9], matches the transductive paradigm. The authors collected gene expression data from the Gene Expression Omnibus (GEO) for inflammatory diseases, consisting of samples labeled either ‘healthy’ or ‘sick’, that had been measured for mouse and human and constructed 36 matched pairs to which they applied various machine learning techniques [e.g. support-vector machines (SVM), k-nearest-neighbor classifiers (kNN), random forests (RF) and neural nets]. The best result in terms of precision and recall for learning the human labels, and, consequently, differentially expressed genes (DEGs, contrasting ‘healthy’ and ‘sick’), and pathways, were obtained by a semi-supervised neural net, which iteratively used the human data to augment the mouse data sets (when validating the method, the ground truth comes from DEGs and pathways that were identified from human data using human labels). Initially, the neural net classifier was exclusively trained on labeled mouse data and used to predict human labels based on human expression data. In the next step, the human samples with the highest classification confidence were used to generate an augmented training set consisting of mouse and human data. After re-training, the classifier was then anew applied to the remaining human data, and again, the samples with the highest classification confidence were incorporated into the cross-species training set. The iteration ended when finally all human data were incorporated and classified. Note that this algorithm does not require the true human labels, the integration works by only using the predicted labels; the true human labels are used for validation. This strategy is a clever way to humanize animal data and seems to be applicable to a wide field of problems and shows high relevance, given the lack of generalization that is often encountered between mouse and human biology. It does, however, require a classification task and is not suitable for regression problems (such as predicting age, speed or other numerical values), since only in the classification case the output of the machine learning algorithm can be used to assign a high or low confidence to the prediction (depending on how much a prediction is ‘between’ classes).

*XGSEA* aims to predict gene sets of interest in a target species under a given condition based on the gene expression of another (source) species under the same or a comparable condition [18]. Shortly, gene set enrichment analysis is performed on the gene expression data of the source species, say mice, comparing a condition of interest to a control and thus determining significantly enriched gene sets (e.g. mouse gene ontology terms) for that condition. The gene sets of the target species (e.g. human gene ontology terms) are then subjected to domain adaptation based on sequence homology between their constituent genes, minimizing divergence between source and target domains while maintaining the pairwise distance between the gene sets across the domains. After domain adaptation, the tool then offers multiple options for transferring gene set enrichment predictions from the source domain to the target domain, using (i) logistic regression to predict the *P*-values of each gene set in the target domain based on those in the source domain; (ii) regression on the enrichment values for each gene set, then calculating the *P*-values directly from those or (iii) regression on the enrichment values for each gene set in each direction (over and under enrichment) before calculating *P*-values as described before. These methods were evaluated against three naïve methods (all three based on mapping target genes to source genes based on sequence homology) in four different datasets, three mouse to human and one zebrafish to human transfer tasks. Generally, XGSEA outperformed the naïve methods for these datasets [evaluated by comparing the area under the receiver operating characteristic (AUROC) at a range of enrichment *P*-value thresholds], in particular when performing regression on enrichment values for each direction of enrichment separately. To test the method further, XGSEA was used to analyze a CD8+ T-cell ATACseq dataset, predicting the enriched pathways in human solid tumors from murine tumor data. The method identified gene expression and immune system terms as likely being enriched in the human tissue. A naïve approach performed on the same data returned a larger number of more diverse terms, so that in this case, XGSEA gave more focused results.

**Figure 2:**
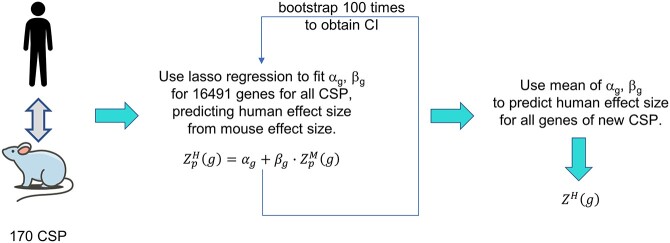
Overview of the FIT algorithm [19]. FIT consists of a compendium of 170 CSP of mouse and human transcriptomics data for 28 different diseases. First, a lasso regression is performed to fit parameters *α* and *β* of a linear model based on all genes, g, of all CSP, p, penalizing *α* values deviating from 0 and *β* values deviating from 1. The fitting process is repeated 100 times to obtain mean values and confidence intervals of the two parameters (see also main text). For a new mouse expression data set, the mean values of the parameters *α* and *β* are then used to predict human effects sizes *Z^H^* for each gene therein. (Mouse clipart by Vincent Le Moign / CC BY 4.0.)

*Found In Translation (FIT)* [19] follows the unsupervised paradigm, aiming to transfer the property of being a high-effect gene from mouse to human, where a high-effect gene is characterized by a high fold change for RNA-seq datasets or a high *z*-test value for microarray datasets. The authors assembled mouse and human gene expression datasets from GEO database that each compared a disease condition versus healthy, created 170 cross species pairings (CSP) spanning 28 human diseases (and the corresponding mouse models) and constructed a model for each CSP that aims to predict human expression values based on mouse expression values according to a linear relationship. The resulting model parameters are used to predict human gene expression from mouse gene expression, in a disease-specific manner, highlighting the high-effect genes. Figure 2 provides an overview of the algorithm. The accuracy of the transfer is estimated from human disease-specific datasets (disjunct from the ones on which the CSP are based) by checking whether the predicted high-effect human genes match already known ones. In fact, the FIT approach increases the number of true-positive predictions of human DEGs from mouse data by 20–50% compared with direct from mouse extrapolation [19]. The smaller the confidence interval of the fitted parameters (Figure 2), the higher the increase of true-positives. Furthermore, it is possible to predict which new mouse data can be extrapolated to humans by FIT using an SVM classifier. The SVM basically tests whether the new mouse data bear enough resemblance to the mouse data of the 170 CSPs or not.

*Translatable Components Regression (TransComp-R)* by [20] presents an application of transfer learning to predict resistance to inflammatory bowel disease treatment with infliximab. The authors aim to transfer knowledge not only from one species to another but also from the space of transcriptomics to proteomics. Labeled human transcriptomics data are used to infer which mouse proteomics data are predictive for responder versus non-responder phenotype in humans. First, human gene expression data are selected for genes associated with the responder phenotype. Next, mouse proteomics data are chosen for genes homologous to the human ones selected in the previous step, and a PCA analysis is performed on these. Finally, the human transcriptomics data are projected into the PCA space and regression against the human responder phenotype is performed, allowing to identify new mouse proteins that might be predictive for the human phenotype. Using this approach, the authors predicted a collagen-binding integrin to be involved in resistance to treatment, a result that was supported *in vitro* (in humans) using anti-integrin antibodies. A limitation of the current approach is that it requires one-to-one mouse–human homologous proteins/genes, but it can be extended to other molecular data and species.

*PROGENy* and *DoRothEA*, in this context, are two approaches specifically aiming to recover perturbations in mice at the pathway (PROGENy) and transcription factor (DoRothEA) level using public human gene expression data. The first tool (PROGENy) was originally developed to assess the activity of human signaling pathways from human gene expression data by finding pathway-specific transcriptomic footprints that entail targets of such pathways [21]. In turn, DoRothEA was initially built to assess associations between transcription factor activities and drug responses in human transcriptomic data, and then it was reformulated as a resource of regulons, i.e. curated transcription factors and their transcriptional targets [22]; these regulons were curated and collected from various public experimental and literature sources and from the GTEx and the TCGA (The Cancer Genome Atlas). Pathway and transcription factor footprints tend to be evolutionarily conserved between humans and mice, and because various studies have demonstrated that it is possible to estimate human gene expression from mouse gene expression data [9, 19], the authors of PROGENy and DoRothEA adapted both tools to work with mouse data, finding 4020 significant associations between pathways and transcription factors in mouse and human diseases by using human–mouse ortholog information. They demonstrated these approaches by estimating the transcription factor and pathway activities from a large collection of mouse *in vitro* experiments, such as chemical and genetic perturbations, as well as from mouse *in vivo* disease-related experiments, and provided these results as an interactive web application. PROGENy and DoRothEA estimate ‘footprints’ of a pathway or a transcription factor on gene expression, and the evolutionary conservation of footprint effects between human and mouse can be further investigated in detail [23]. On this basis, disease associations and perturbations can be inferred (and validated, e.g. by checking human-based predictions in mice), alongside pathway and transcription factor activity scores for a large collection of human and mouse perturbation and disease experiments.

*Adversarial Inductive Transfer Learning (AITL)* [12] is explicitly described using the terminology of [13], and it bridges *in vitro* (source domain, human cancer cell line data) and *in vivo* (target domain, human cancer patient data) in two ways. Firstly, it transfers gene expression knowledge based on cell-lines to patients, where the expression profiling was done to describe the response to chemotherapeutic drugs. For cell lines, the data stem from the GDSC (Genomics of Drug Sensitivity in Cancer) database, and the labels are IC50 values. For patients, the data stem from the TCGA and some other sources, and the labels (if available) are binary, reflecting response/non-response to chemotherapy (yes/no). Then, the different output labels are handled by multi-task learning. More specifically, a multi-class predictor is trained on both source and target samples, utilizing a binarized outcome in the case of the source samples; this simultaneous learning on the source and target data is also suggested to improve accuracy. The ‘biological’ differences in the gene expression input data are handled by adversarial domain adaptation. In more detail, shared denominators (called ‘extracted features’ in the AITL framework) are learned in a domain-invariant manner by employing an adversary network tasked with distinguishing the domains; its failure is rewarded. If the extracted features learned by AITL play a similar role in both source and target domains, AITL transfer learning can be successful. AITL was benchmarked against state-of-the-art methods such as PRECISE [24] (see below), ADDA [25], MOLI [26], ProtoNet [27, 28] and [29] based on AUROC and area under the precision-recall curve. In all experiments, AITL performed better. AITL is especially relevant for small clinical sample sizes as encountered in pharmacogenomics. Even though AITL was only used for gene expression data, it could be extended to multi-omics scenarios.

*PRECISE* (Patient Response Estimation Corrected by Interpolation of Subspace Embeddings) [24] uses preclinical models (cell lines and patient-derived xenografts) as predictors, despite their inherent differences compared with real human tumors. To identify common molecular mechanisms (based on similarity of gene expression) in preclinical models and human tumors, PRECISE processes transcriptomic data to first find specific underlying ‘factors’ (based on a PCA) for each set (preclinical models and human tumors) separately, and the factors from both sets are then aligned and compared, to generate common factors (or principal vectors) between both sets, the most similar of which are then used to generate a consensus representation of the tumor model. This consensus representation is then finally employed to train a regression model of the preclinical gene expression data with respect to the preclinical drug response data, which is then applied to the real human tumor gene expression data to predict human tumor response. Despite the superior performance of the method compared with state-of-the-art work, the study only applied its framework to gene expression data while acknowledging the benefits of multiomics-based approaches.

**Figure 3:**
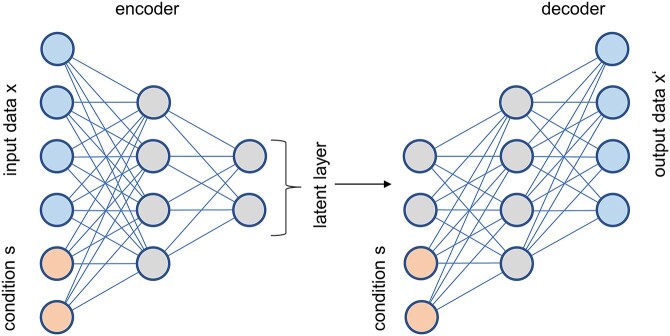
Overview of the trVAE. The encoder part of the neural net processes input data plus information about the condition (smiling, cell type, etc.) and generates a compressed latent layer. The decoder uses this latent layer together with information about the condition to produce an output. During training, the condition fed to the encoder and the decoder are the same, while during prediction, the decoder receives the new condition for which an output should be generated. In contrast to a standard conditional VAE, an additional constraint is imposed on the first layer of the decoder for further regularization, the details of which we omitted here. While two input nodes are needed for the condition if an unary (‘one-hot’) encoding is used, the number of nodes in the other parts of the neural nets is far larger than shown here, for any realistic application. The diagram is redrawn from [30].

*Conditional Out-of-Distribution Transfer Learning* [30] employs a transfer variational autoencoder (trVAE), which enables the transfer of conditions across domains. This makes it for instance possible to train a neural net on images of smiling and non-smiling men and non-smiling women and then transfer the smiling condition (‘style’) from the male to the female domain. Similarly, the authors applied trVAE to a single-cell gene expression dataset of the gut (comprising eight different cell types) after infections with different bacteria, obtained from public data sources published in other papers. The method could successfully transfer the effects of the infection to cell types not included in training. As the name implies, the architecture of trVAE is based on an autoencoder, an unsupervised neural net where the output layer is trained to reproduce the input layer while going through a bottleneck layer in between. trVAE modifies this approach by explicitly providing the first decoder layer with information about the condition of the input sample (e.g. smiling versus non-smiling). During training, all samples are supplied with their correct condition, but for prediction the desired condition (e.g. smiling) is used as extra input to the decoder, causing the last layer of the decoder to contain a representation of the input modified by the desired condition. For a better understanding of the algorithm, we provide an overview in Figure 3. The authors benchmarked trVAE against standard CVAE [31], MMD-CVAE (similar to VFAE) [32], MMD-regularized autoencoder [33, 34], CycleGAN [35], scGen [36] and scVI [37] by comparing Pearson’s correlation values for mean and variance of gene expression and found that trVAE performed best. Thus, tools like trVAE might be used to make predictions about human tissues from which no biopsy can be obtained (e.g. brain) as long as data are available for another tissue (e.g. blood) and from another domain (e.g. brain and blood of mice), enabling clinical outcome predictions based on preclinical data. In principle, trVAE can be used for many kinds of medical data, not just omics or image data.

**Figure 4:**
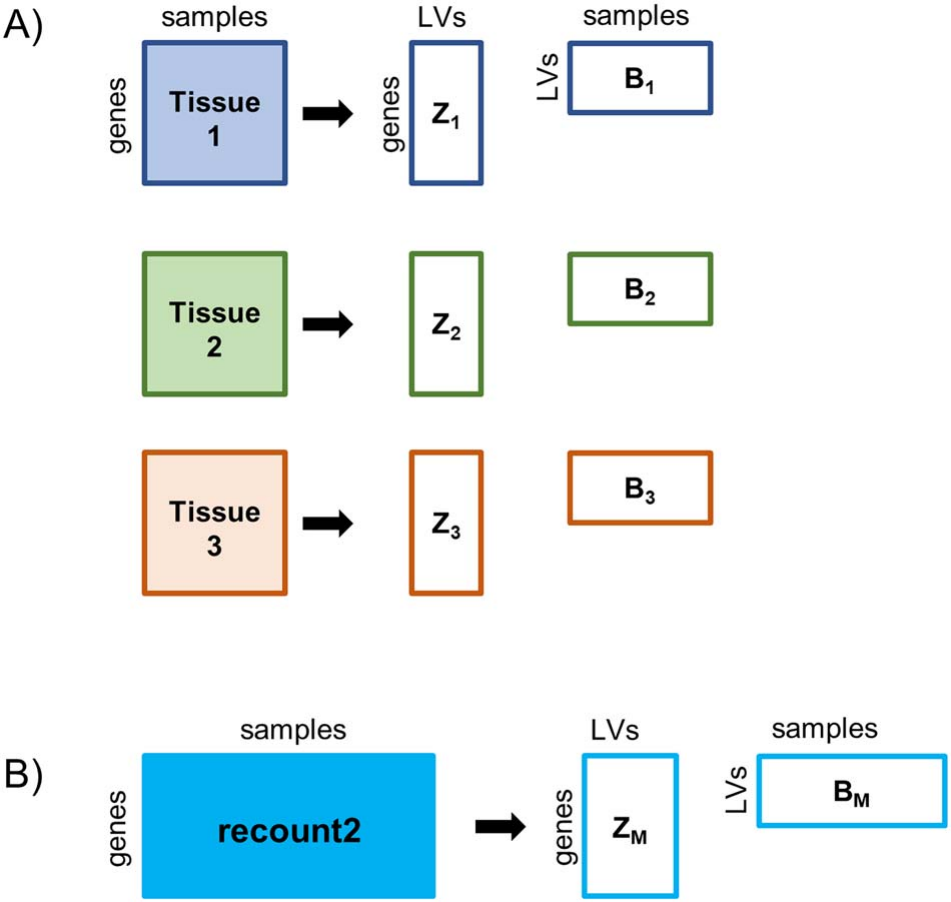
Overview of MultiPLIER framework. (**A**) PLIER [38], on which MultiPLIER is based, can analyze tissue-specific expression data and extract LVs by matrix factorization, resulting in matrices *B* and *Z*. PLIER then aligns the LVs with curated pathway gene sets in a downstream analysis. (**B**) To analyze data irrespective of tissue, MultiPLIER trains on a large collection of uniformly processed data in the form of the recount2 compendium [39], which contains around 370 000 samples. The resulting LVs can then be used to interpret a new dataset, by projecting the new gene expression data onto the latent space, to identify pathway-annotated LVs also featured in that new dataset. Diagram is based on [15].

*MultiPlier* [15] is an unsupervised learning approach aiming to transfer feature representations (LVs, i.e. ‘patterns’ based on correlation of gene expression calculated by PLIER [38]) from the source to the target domain. The source domain is derived from recount2 [39], a collection of disease-related gene expression datasets generated by next-generation sequencing (NGS), where all raw data were processed in a unified way, reflecting a wide variety of biological processes and pathways based on gene expression of multiple tissues and diseases. The target domain can entail any gene expression dataset that is expected to feature at least some of these processes; rare disease datasets are the use case, because they feature few samples (almost) by definition. The LVs (‘patterns’ of correlated genes) are calculated for the source by matrix factorization, so that LVs partly associate with some known pathways or cell-type-specific gene sets. Once the LVs are learned, a new gene expression dataset can be projected into the space defined by these, and the authors show that this projection is effective in revealing biological processes related to rare disease severity. Even though the approach addressed rare disease, the method could also benefit common diseases by stratification of responsive subgroups. Moreover, even though the model was based on multiple diseases, these were all related to diseases involving auto-immune components [40]. Also, some performance issues were discussed, including the correlation of LVs with the biological factors [40]. Figure 4 provides a description of the MultiPLIER framework. Most recently, LVs derived by MultiPlier were used as input features to classify subtypes of cancer using RF [41].

## Discussion, conclusion and perspectives

The ultimate goal in biomedical research is to understand and tackle a disease or dysfunction in humans. What are the molecular foundations of a certain disease? Which are the diagnostic, prognostic or predictive biomarkers? Is a certain intervention effective in humans? What are the pharmacokinetics and pharmacodynamics of a drug? Unfortunately, it is often not possible to perform the necessary experiments and measurements in humans for ethical, financial or technical reasons or because access to existing clinical data is limited due to data protection and other issues. Also, measuring longitudinal outcomes may simply take too long, given the long lifespan of humans. For these reasons, researchers use alternative model systems in the hope that the insights from those systems can be applied to humans, yet, due to the limited generalizability of such alternative model systems, conclusions drawn can lead to failures. Accurate transfer learning is expected to improve this situation by extracting the shared denominators of the two domains, preclinical and clinical.

We assigned all transfer learning methods reviewed to one of the three fundamental kinds of transfer learning, that is, inductive, transductive or unsupervised. For these three kinds of transfer learning, we found a variety of application areas, as follows. Transfer from species to species is important because animals are usually short-lived and allow experiments under controlled conditions that are not possible in humans. The more closely related the species is to humans, the more likely the transfer is expected to succeed, given an appropriate transfer learning approach. ‘Found in Translation’ [19] is specifically designed to transfer results from mouse to human for 28 disease models. Similarly, the semi-supervised method of [9] also transfers between mice and humans, where the authors focus on inflammatory diseases (other diseases were not investigated). In both cases, gene/protein homology information is needed. A second relevant area of transfer is from one omics to another, specifically from transcriptomics to proteomics, as shown in this review. With modern NGS techniques, transcriptomics data can easily be measured, but one would like to infer information about the proteome since proteins are the biomechanical machines that eventually perform most tasks in the cell. TransComp-R [20] is such a tool that in addition also transfers information between species (mouse and human). *In -vitro* studies are also used to approximate the human *in vivo* situation. Thus, *in vitro* to *in vivo* transfer is another important area for transfer learning. AITL [12] is one such example, which transfers knowledge from the *in vitro* to the *in vivo* situation (and from quantitative output to a binary output). Not surprisingly, the tasks are different because the biology is not the same in cells versus humans, and inductive learning must be done. Finally, it is very helpful to transfer knowledge from tissue to tissue. In humans, it is often not possible to obtain a sample from the affected tissue (e.g. brain or pancreas), but a blood sample can be collected easily and non-invasively. Moreover, the flow of blood connects most tissues of the body in one way or another, so we can expect to find traces of many organ-specific processes in the blood. Also, in cancer, for example, disease-specific nucleic acids can be traced and monitored in blood samples. Using the blood transcriptome as a proxy for disease processes in other organs opens the way for a personalized medicine approach that is complementary to genetics-based approaches. For transfer learning from tissue to tissue, trVAE may be employed.

For molecular data, some structuring of the shared denominators may enhance the success rates of transfer learning. For one, deep neural-net-based learning is essentially a black-box approach frequently employed in transfer learning. However, structuring neural nets based on hierarchical knowledge (such as the gene ontology, GO) gained momentum and acceptance [42, 43], and ontologies may be a key to structure the space of shared denominators yielding not just better accuracy in predictor performance, but also better interpretability of the prediction/classification process. Here, knowledge about master regulators and (signaling) pathways may be encoded by gene/protein interaction and regulation subnetworks, which may enable an even better structuring than the GO hierarchy, and in fact, the development of the GO is heading in a similar direction, towards investigating GO-Causal-Activity-Models [44].

It is important to assess the generalization ability of predictors derived by transfer learning, that is, their accurate performance on unseen datasets. Extensive validation on samples processed with different platforms is also important. For human studies, validating on an unseen cohort allows estimating generalization capacity by comparison to the original prediction error [45]. Also, the comparison to the results of other state-of-the-art approaches is useful for judging the accuracy of a transfer method. Another useful consideration is the correlation of the shared denominators with biological knowledge. Not addressed by any of the methods, but a highly relevant complementary aspect, is the use of explainability methods [46] to understand and consecutively improve transfer learning methods and results.

In this review, we provided a sample of available prediction tools and algorithms for transfer learning in biomedicine; yet, there are a large number of approaches and software packages. It is quite challenging to adequately compare all these tools in a coherent and fair way, but we hope to have provided a starting point. Moreover, we hope that Table 1, Figure 1 and the text may be of help to anyone facing a learning task that may profit from transfer learning.

**Key Points**

- Transfer learning in biomedicine is gaining momentum, reflecting the scarcity of human data (target domain) compared to animal and *in vitro* data (source domain).
- Shared denominators (e.g. latent variables) enable the accurate transfer of predictors from the source to the target domain.
- Examples of unsupervised, supervised inductive and supervised transductive transfer learning are described and tabulated.
- A basis is provided to guide the user regarding the selection of the type of transfer learning most appropriate for a new task.

## Glossary

*In vivo*: a process or experiment that occurs in a living organism, e.g. clinical intervention trials.

*Ex vivo*: a process or experiment that occurs outside a living organism but with minimal changes, e.g. experiments on tissue samples recently taken from a living subject.

*In vitro*: a process or experiment that occurs outside a living organism, isolating the sample from its typical biological context, e.g. cell culture experiments using established cell lines.

Phenotype: the observable characteristics of an organism.

Biomarker: a measurable biological feature that can be used to effectively predict another (often more complex) biological feature of interest.

Transcription factor: a DNA-binding protein involved in the regulation of the rate of transcription of a gene or a set of genes.

## Author Biographies

**Axel Kowald** is a postdoctoral researcher at the Rostock University Medical Center. His research is focused on bioinformatics and systems biology with respect to elucidating the aging process.

**Israel Barrantes** is a postdoctoral researcher at the Rostock University Medical Center. His research is focused on the analysis of high-throughput data related to infection research and clinical and environmental microbiomes.

**Steffen Möller** is a postdoctoral researcher at the Rostock University Medical Center. His research is focused on developing bioinformatics tools and intergenomic analyses of genetic and RNA expression data.

**Daniel Palmer** is a postdoctoral researcher at the Rostock University Medical Center. His research is focused on the analysis of clinical and high-throughput data.

**Hugo M. Escobar** is a senior scientist at the Hematology, Oncology and Palliative Medicine, Rostock University Medical Center. His primary research area is in molecular oncology.

**Anne Schwerk** works on machine learning at the CENTOGENE GmbH in Rostock.

**Georg Fuellen** is a professor of medical bioinformatics at the Rostock University Medical Center.
